# Antioxidant Systems from Pepper (*Capsicum annuum* L.): Involvement in the Response to Temperature Changes in Ripe Fruits

**DOI:** 10.3390/ijms14059556

**Published:** 2013-05-02

**Authors:** Rosa M. Mateos, Ana Jiménez, Paloma Román, Félix Romojaro, Sierra Bacarizo, Marina Leterrier, Manuel Gómez, Francisca Sevilla, Luis A. del Río, Francisco J. Corpas, José M. Palma

**Affiliations:** 1 Department of Biochemistry, Cell and Molecular Biology of Plants, Estación Experimental del Zaidín, CSIC, Apartado 419, Granada E-18080, Spain; E-Mails: promanfarmacia@msn.com (P.R.); leterriermarina@yahoo.fr (M.L.); luisalfonso.delrio@eez.csic.es (L.A.R.); javier.corpas@eez.csic.es (F.J.C.); 2 Department of Stress Biology and Plant Pathology, Centro de Edafología y Biología Aplicada del Segura, CSIC, Apartado 164, Murcia E-30100, Spain; E-Mails: ajimenez@cebas.csic.es (A.J.); felix@cebas.csic.es (F.R.); fsevilla@cebas.csic.es (F.S.); 3 Syngenta Seeds, S.A., El Ejido E-04710, Almería, Spain; E-Mail: sierra.bacarizo@syngenta.com; 4 Estación Experimental del Zaidín, CSIC, Apartado 419, Granada E-18080, Spain; E-Mail: manuelgomez@cofgranada.com

**Keywords:** antioxidative enzymes, ascorbate, *Capsicum annuum* L., gene expression, lipid peroxidation, NADP-dehydrogenases, pepper fruit, protein oxidation, temperature changes, western blotting

## Abstract

Sweet pepper is susceptible to changes in the environmental conditions, especially temperatures below 15 °C. In this work, two sets of pepper fruits (*Capsicum annuum* L.) which underwent distinct temperature profiles *in planta* were investigated. Accordingly, two harvesting times corresponding to each set were established: Harvest 1, whose fruits developed and ripened at 14.9 °C as average temperature; and Harvest 2, with average temperature of 12.4 °C. The oxidative metabolism was analyzed in all fruits. Although total ascorbate content did not vary between Harvests, a shift from the reduced to the oxidized form (dehydroascorbate), accompanied by a higher ascorbate peroxidase activity, was observed in Harvest 2 with respect to Harvest 1. Moreover, a decrease of the ascorbate-generating enzymatic system, the γ-galactono-lactone dehydrogenase, was found at Harvest 2. The activity values of the NADP-dependent dehydrogenases analyzed seem to indicate that a lower NADPH synthesis may occur in fruits which underwent lower temperature conditions. In spite of the important changes observed in the oxidative metabolism in fruits subjected to lower temperature, no oxidative stress appears to occur, as indicated by the lipid peroxidation and protein oxidation profiles. Thus, the antioxidative systems of pepper fruits seem to be involved in the response against temperature changes.

## 1. Introduction

Pepper (*Capsicum annuum* L.) is the second-most consumed vegetable worldwide and is characterized by its high levels of vitamin C (ascorbic acid), pro-vitamin A (carotene) and calcium. In fact, intakes of 50–100 g fresh pepper fruits could provide 100% and about 60% of the recommended daily amounts of vitamin C and A, respectively [1–5]. Mature pepper fruits are also rich in carotenoids, compounds with antioxidant and anti-carcinogenic capacity; furthermore, either immature or mature fruits contain a high concentration of antioxidant phenolic compounds [1,6]. Much of the information available on the antioxidative features of pepper fruits has been obtained from investigations addressed to elucidate the influence of pepper consumption on the human health due to the fruit quality [7–10]. However, little is still known on the role of antioxidants on the fruit metabolism under temperatures below the optimal physiological requirements.

Based on some organoleptic features and culinary purposes, pepper fruits are usually classified into two types. The term bell pepper is used to refer to a non-pungent, chunky sweet pepper type, whereas chili pepper generally refers to pungent chili fruits [11]. Based on the shape, bell/sweet peppers are mainly classified in types California, Lamuyo and Dulce italiano (see Figure 9 from Experimental Section 4.1). California fruits used in this work commonly shift to red/yellow/orange color after maturation, and are characterized by similar sizes of the transversal and longitudinal axes. So, these fruits usually correspond to “square” peppers (*Capsicum* in Latin means box). In most *Capsicum* species, ripening is characterized by a very intense metabolism, emitting volatile organic compounds associated to respiration, with destruction of chlorophyll, synthesis of new pigments (red/yellow carotenoids plus related xanthophylls, anthocyanins), formation of pectins, protein synthesis, taste alteration as a consequence of modification in acidity, pH and astringency, and changes in total soluble reducing equivalents [1,12–15].

Pepper plants are originally from tropic regions and require high temperature conditions for their development. Consequently, the optimum growth temperature is between 25 and 30 °C, in such a way that temperature changes affect a variety of physiological functions and morphological development. When temperature decreases below 15 °C, pepper growth is reduced, and bloom and fruit production stop [16]. Low temperature affects pepper vegetative development and reproduction by disturbing the function of the flower female organs and the number of viable pollen grains per flower [17–21]. Thus, fruits from plants that have been set under low night temperatures (14 °C or less) usually are deformed and seedless causing significant economical losses.

Under stress conditions and senescence in plants, an increase in the generation of reactive oxygen species (ROS) usually takes place in different cell compartments, including superoxide radicals (O_2_^·−^), hydrogen peroxide (H_2_O_2_) and hydroxyl radicals (^·^OH) [22–32]. These species can react with proteins, lipids and nucleic acids altering the biological potentiality of these biomolecules [33]. In addition to this, plants are especially susceptible to the oxidative damage produced by ROS [34–38]. The cellular oxidative damage plays an important role in determining the relative efficiency of the cell function and, therefore, the behavior of cultures (crops) under changing environmental conditions.

Cells from higher plants contain antioxidant systems that are able to scavenge ROS, thus allowing the cell to cope with different stress situations. The main antioxidant enzymatic systems are catalase, superoxide dismutase (either CuZn-SOD, Fe-SOD or Mn-SOD), and the ascorbate-glutathione cycle (AGC) which includes the enzymes ascorbate peroxidase (APX), monodehydroascorbate reductase (MDAR), dehydroascorbate reductase (DAR) and glutathione reductase (GR) [33,35,39–43]. Also, several NADPH generating enzymes, which provide reducing power to the cell metabolism, are considered as antioxidant enzymes, and these include glucose-6-phosphate dehydrogenase (G6PDH), 6-phosphogluconate dehydrogenase (6PGDH), isocitrate dehydrogenase (ICDH) and the malic enzyme (ME) [44–47]. Among the low-molecular-weight non-enzymatic antioxidants the most important in plants are ascorbate (vitamin C), glutathione, α-tocopherol (vitamin E), flavonoids and carotenoids [25,33].

Current breeding and marketing strategies of crop vegetables such as pepper emphasize on the search of molecular indexes which could be used to select cultivars with enhanced tolerance to environmental stress such as temperature changes (decreases). Thus, considering the nutritional and socio-economical relevance of pepper, a better knowledge of the molecular changes responsible for the fruit physiology under climate changes could supply valuable information to be used to obtain varieties with improved quality.

## 2. Results

**Figure 1:**
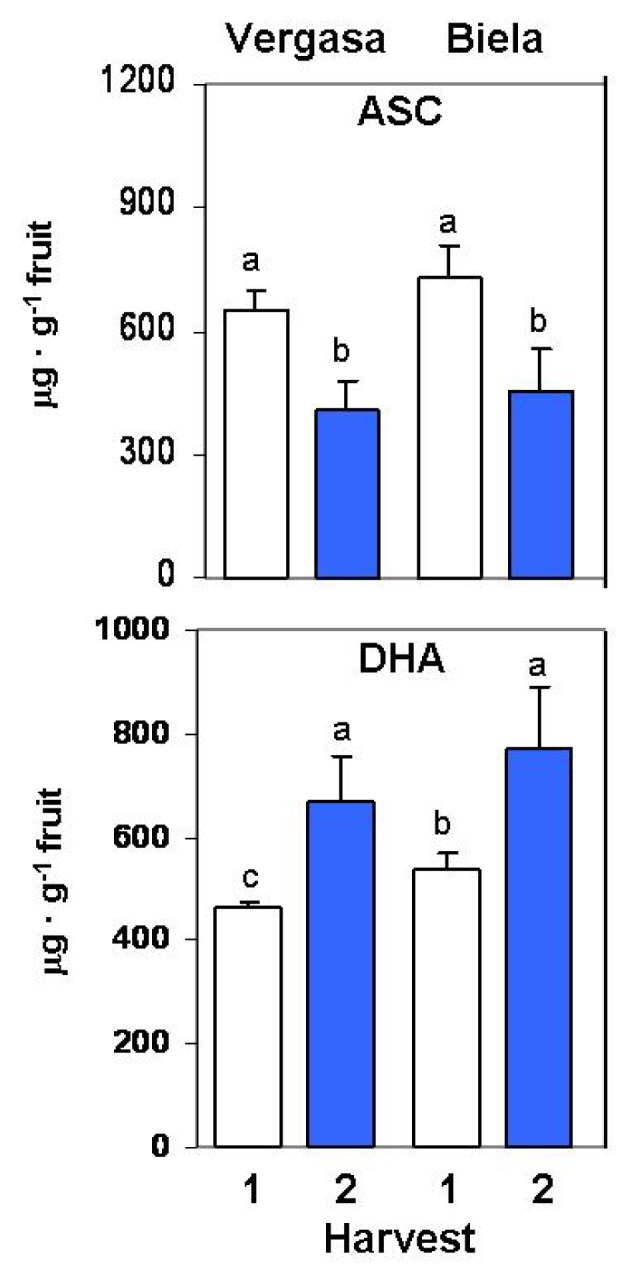
Ascorbate (ASC) and dehydroascorbate (DHA) contents in cultivars Vergasa and Biela of pepper (*Capsicum annuum* L.) fruits harvested at two temperature conditions (Harvest 1 and 2). Column values with distinct letters are significantly different.

In this work fruits from two cultivars (Vergasa and Biela) of the California-type pepper with different ripening pattern were used (see Experimental Sections for phenotype characteristics) in order to prove whether the response against temperature change episodes was homogenous in these cultivars from the same pepper type or it depended on the metabolic features of each one. The analysis of the ascorbate content in the two cultivars and the two harvests set for this study showed that the reduced ascorbate form (ASC) was lowered in both cutivars at Harvest 2, whereas the oxidized form (dehydroascorbate; DHA) was enhanced (Figure 1). On the other hand, in spite of the differences observed in the ASC and DHA contents, the total ascorbate pool did not vary in any of the two cultivars, and the ratio ASC/DHA was double at Harvest 1 (Table 1).

The total antioxidant activity (TAA) was determined in all the samples by the ABTS method, and no important changes were detected in the fruits between the two harvesting times for the two cultivars used in this work (Table 1). The analysis of the protein concentration in fruits revealed important increases at Harvest 2, the main changes being observed in cultivar Biela which doubled its protein content (Table 2).

**Figure 2:**
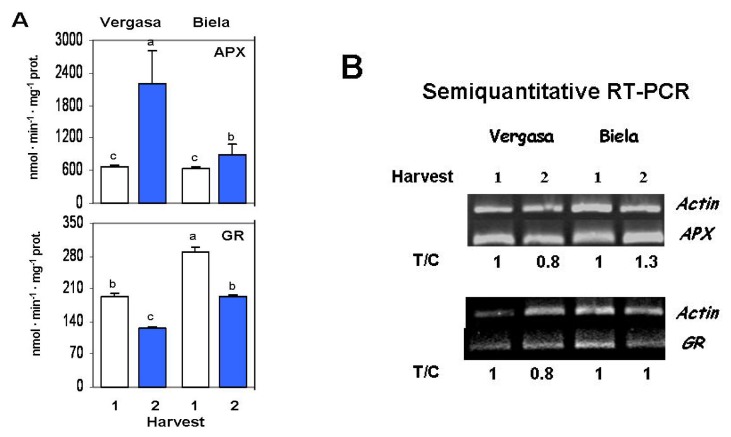
Activity and expression levels of the ascorbate-glutathione cycle enzymes in the cultivars Vergasa and Biela of pepper (*Capsicum annuum* L.) fruits harvested at two temperature conditions (Harvest 1 and 2). (**A**) Activity of ascorbate peroxidase (APX) and glutathione reductase (GR) determined spectrophotometrically. Column values with distinct letters are significantly different; (**B**) Analysis of the mRNA expression. Semiquantitative reverse transcription-PCR was performed on total RNA isolated from fruits of the two cultivars. A representative agarose electrophoresis gel of the amplification products visualized by ethidium bromide staining under UV light is shown. T/C indicates the relative level of the *APX* and *GR* amplification products (T) over the *Actin* (C, internal control) after normalization to the control samples and expresses the change in folds with respect to the corresponding controls (*Actin* from Harvest 1 in each cultivar). Values are means of at least three semiquantitative RT-PCR assays made in different fruits from each cultivar.

Some of the ascorbate-glutathione cycle components were studied in the plant materials. Ascorbate peroxidase (APX) activity increased in Vergasa and Biela at Harvest 2 with respect to Harvest 1 (Figure 2A). The analysis of a specific *APX* gene level by semiquantitative RT-PCR showed that only in Biela the transcript levels slightly increased in Harvest 2 (Figure 2B). Glutathione reductase activity was lower in Harvest 2 from the two cultivars studied in this work (Figure 2A). Nevertheless, the expression level of a specific *GR* gene was unmodified between harvests in both cultivars (Figure 2B).

**Figure 3:**
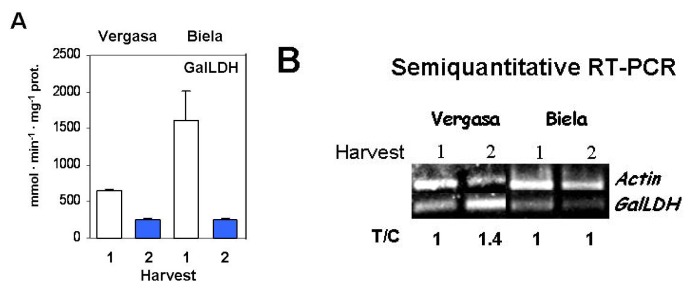
l-Galactono-γ-lactone dehydrogenase (GalLDH) activity and expression level in the cultivars Vergasa and Biela of pepper (*Capsicum annuum* L.) fruits harvested at two temperature conditions (Harvest 1 and 2). (**A**) Activity determined spectrophotometrically. Column values with distinct letters are significantly different; (**B**) Analysis of the mRNA expression. Semiquantitative reverse transcription-PCR was performed on total RNA isolated from fruits of the two cultivars. A representative agarose electrophoresis gel of the amplification products visualized by ethidium bromide staining under UV light is shown. T/C indicates the relative level of the *GalLDH* amplification product (T) over the *Actin* (C, internal control) after normalization to the control samples and expresses the change in folds with respect to the corresponding controls (*Actin* from Harvest 1 in each cultivar). Values are means of at least three semiquantitative RT-PCR assays made in different fruits from each cultivar.

To investigate the potential ascorbate synthesis in the fruits, the activity and expression levels of GalLDH were analyzed (Figure 3). Although both cultivars displayed a greater activity at Harvest 1, this was much higher in cultivar Biela (Figure 3A). At the present moment it is still early to understand this behavior in this cultivar whose most prominent peculiarity is its color shift from green to yellow at ripening. More research is needed to clarify this concern. Regarding to the *GalLDH* gene expression, some fluctuations were detected. Thus, whereas in Vergasa transcript increases of about 40% were found, the transcript levels in Biela remained constant (Figure 3B).

**Figure 4:**
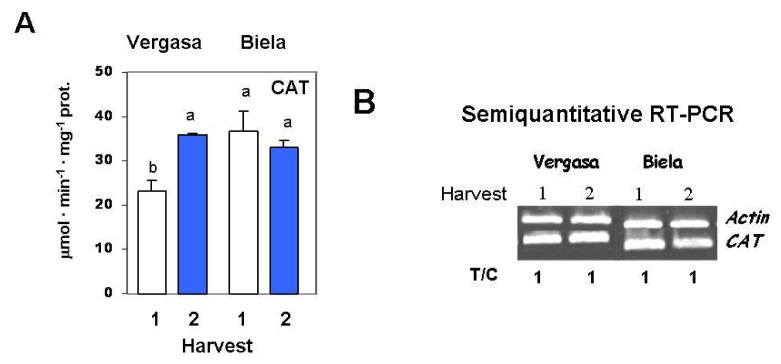
Catalase (CAT) activity and expression level in cultivars Vergasa and Biela of pepper (*Capsicum annuum* L.) fruits harvested at two temperature conditions (Harvest 1 and 2). (**A**) Activity determined spectrophotometrically. Column values with distinct letters are significantly different; (**B**) Analysis of mRNA expression. Semiquantitative reverse transcription-PCR was performed on total RNA isolated from fruits of the two cultivars. A representative agarose electrophoresis gel of the amplification products visualized by ethidium bromide staining under UV light is shown. T/C indicates the relative level of the *CAT* amplification product (T) over the *Actin* (C, internal control) after normalization to the control samples and expresses the change in folds with respect to the corresponding controls (*Actin* from Harvest 1 in each cultivar). Values are means of at least three semiquantitative RT-PCR assays made in different fruits from each cultivar.

Two important antioxidative enzymatic systems were also studied in this work, catalase and superoxide dismutase. Catalase (CAT) activity considerably increased in Vergasa at Harvest 2, whereas Biela did not show differences between harvests (Figure 4A). The expression of *catalase* gene levels was studied in the fruits of the two pepper cultivars by semiquantitative RT-PCR using specific primers from pepper, and it did not show changes at the two selected harvesting times (Figure 4B).

**Figure 5:**
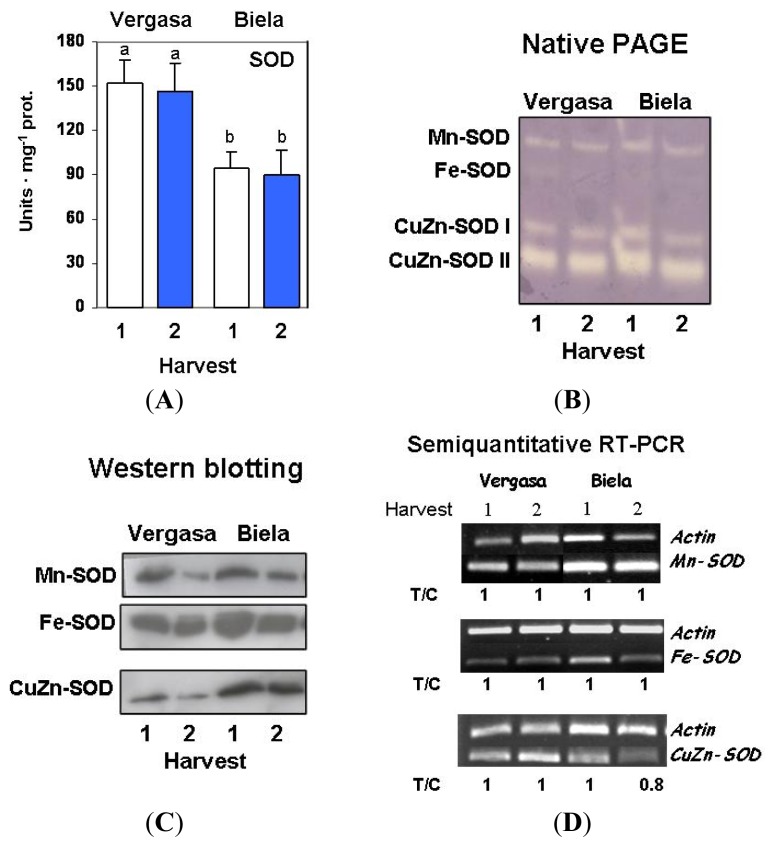
Activity, protein content and expression levels of superoxide dismutase (SOD) in cultivars Vergasa and Biela of pepper (*Capsicum annuum* L.) fruits harvested at two temperature conditions (Harvest 1 and 2). (**A**) Total activity determined spectrophotometrically. Column values with distinct letters are significantly different; (**B**) Isoenzymatic pattern of SOD in the two cultivars where four isozymes were detected. Samples (100 μg protein/lane) were loaded and polyacrylamide gel electrophoresis was carried out under native conditions. Isozymes were characterized on the basis of their susceptibility to the inhibitors KCN and H_2_O_2_; (**C**) Western blotting of samples from the two pepper cultivars using specific antibodies against Mn-SOD, Fe-SOD and CuZn-SOD (see Experimental Section). Prior to transferring proteins (15 μg/lane) to PDVF membranes, polypeptides were separated in 12% polyacrylamide gels under denaturing conditions; (**D**) Analysis of the mRNA expression. Semiquantitative reverse transcription-PCR was performed on total RNA isolated from fruits of the two cultivars. A representative agarose electrophoresis gel of the amplification products visualized by ethidium bromide staining under UV light is shown. T/C indicates the relative level of the *Mn-SOD*, *Fe-SOD* and *CuZn-SOD* amplification products (T) over the *Actin* (C, internal control) after normalization to the control samples and expresses the change in folds with respect to the corresponding controls (*Actin* from Harvest 1 in each cultivar). Values are means of at least three semiquantitative RT-PCR assays made in different fruits from each cultivar.

The superoxide dismutase (SOD) enzymatic system was studied by different approaches. Total SOD activity did not vary with time in any of the two cultivars, but it was higher in Vergasa (Figure 5A). However, neither qualitative nor quantitative changes were observed in the SOD isoenzymatic pattern in fruits from both cultivars at the two harvests (Figure 5B). Four isozymes were detected in crude extracts from pepper fruits: one Mn-SOD, one Fe-SOD, and two CuZn-SODs, designated as CuZn-SOD I and II, according to their increasing electrophoretic mobility in non-denaturing gels. CuZn-SOD II was the most abundant isozyme, whereas Fe-SOD was the minor isozyme in all crude extracts. The protein content of the SOD isozymes was analyzed by western blotting using specific antibodies. In the two cultivars a lower content of the Mn-SOD protein content was observed at Harvest 2 (Figure 5C). In contrast, neither the Fe-SOD nor the CuZn-SOD protein contents seemed to be greatly modified by the time. Regarding to the transcript levels of *Mn-SOD*, *Fe-SOD*, and *CuZn-SOD*, analyzed by using specific primers, no changes were observed for the expression of the *SOD* genes investigated (Figure 5D).

**Figure 6:**
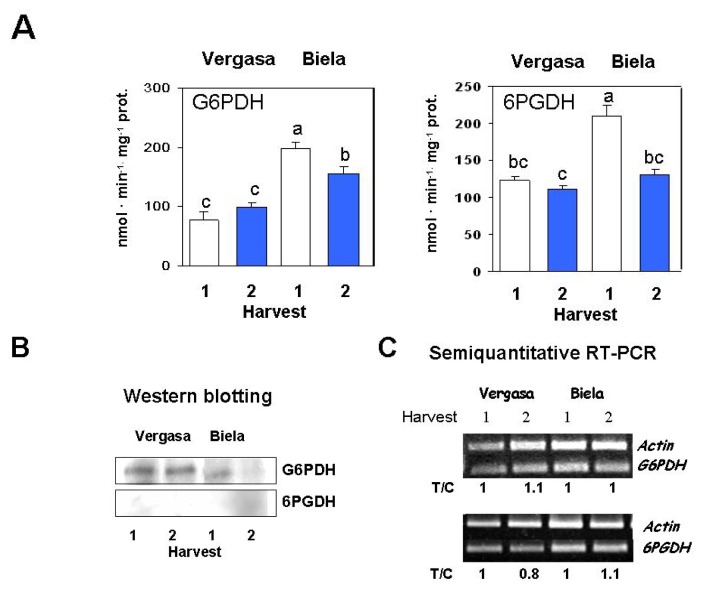
Activity, protein content and expression levels of the glucose-6-phosphate dehydrogenase (G6PDH) and 6-phosphogluconate dehydrogenase (6PGDH) in cultivars Vergasa and Biela of pepper (*Capsicum annuum* L.) fruits harvested at two temperature conditions (Harvest 1 and 2). (**A**) Total activity determined spectrophotometrically. Column values with distinct letters are significantly different; (**B**) Western blotting of samples from the two pepper cultivars using specific antibodies against G6PDH and 6PGDH (see Experimental Section below). Previous to transferring proteins (15 μg/lane) to PDVF membranes, polypeptides were separated in 12% polyacrylamide gels under denaturing conditions; (**C**) Analysis of the mRNA expression. Semiquantitative reverse transcription-PCR was performed on total RNA isolated from fruits of the two cultivars. A representative agarose electrophoresis gel of the amplification products visualized by ethidium bromide staining under UV light is shown. T/C indicates the relative level of the *G6PDH* and *6PGDH* amplification products (T) over the *Actin* (C, internal control) after normalization to the control samples and expresses the change in folds with respect to the corresponding controls (*Actin* from Harvest 1 in each cultivar). Values are means of at least three semiquantitative RT-PCR assays made in different fruits from each cultivar.

The metabolism of some NADPH-generating dehydrogenases was also studied in the selected cultivars. A decrease of G6PDH activity took place at Harvest 2 in Biela, whereas in Vergasa, no significant changes were observed between harvests (Figure 6A). This profile coincided with the specific protein amount for each sample, according to the data obtained by western blotting using antibodies against yeast G6PDH. In cultivar Biela a slight reduction in the intensity of the antibody-recognition bands was observed (Figure 6B). 6PGDH showed a similar pattern to that observed in G6PDH, with activity decreases only observed in Harvest 2 from cultivar Biela (Figure 6A). In the western blotting analysis, negligible bands were detected in the Vergasa and Biela fruits (Figure 6B). With respect to the expression levels of some of the genes coding for G6PDH and 6PGDH, no apparent changes were observed among cultivars and harvests (Figure 6C).

**Figure 7:**
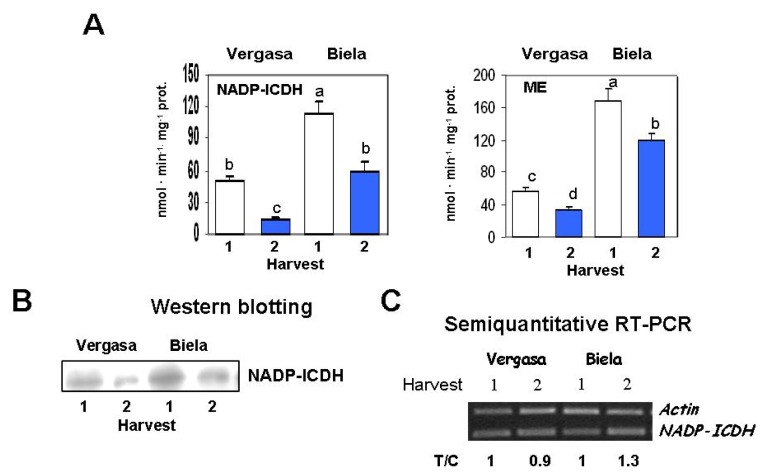
NADP-isocitrate dehydrogenase (NADP-ICDH) and malic enzyme (ME) in cultivars Vergasa and Biela of pepper (*Capsicum annuum* L.) fruits harvested at two temperature conditions (Harvest 1 and 2). (**A**) Total activity determined spectrophotometrically. Column values with distinct letters are significantly different; (**B**) Western blotting of samples from the two pepper cultivars using specific antibodies against NADP-ICDH (see Experimental Section below). Previous to transferring proteins (15 μg/lane) to PDVF membranes, polypeptides were separated by electrophoresis in 12% polyacrylamide gels under denaturing conditions; (**C**) Analysis of the mRNA expression. Semiquantitative reverse transcription-PCR was performed on total RNA isolated from fruits of the two cultivars. A representative agarose electrophoresis gel of the amplification products visualized by ethidium bromide staining under UV light is shown. T/C indicates the relative level of the *NADP-ICDH* amplification product (T) over the *Actin* (C, internal control) after normalization to the control samples and expresses the change in folds with respect to the corresponding controls (*Actin* from Harvest 1 in each cultivar). Values are means of at least three semiquantitative RT-PCR assays made in different fruits from each cultivar.

The analysis of the NADP-ICDH activity showed a common pattern in the two cultivars, with a decline at the second Harvest (Figure 7A). The specific protein content, analyzed by western blotting using an antibody against the pea enzyme, displayed a correlation with the activity pattern with less intense bands in Harvest 2 in both cultivars (Figure 7B). At the gene expression level using specific primers for *NADP-ICDH* (Figure 7C), our results showed a variable pattern with an increase of about 30% in Harvest 2 in the Biela cultivar. ME activity was also reduced in Harvest 2 in the two cultivars studied in this work, with the highest activity being observed in Biela (Figure 7A).

**Figure 8:**
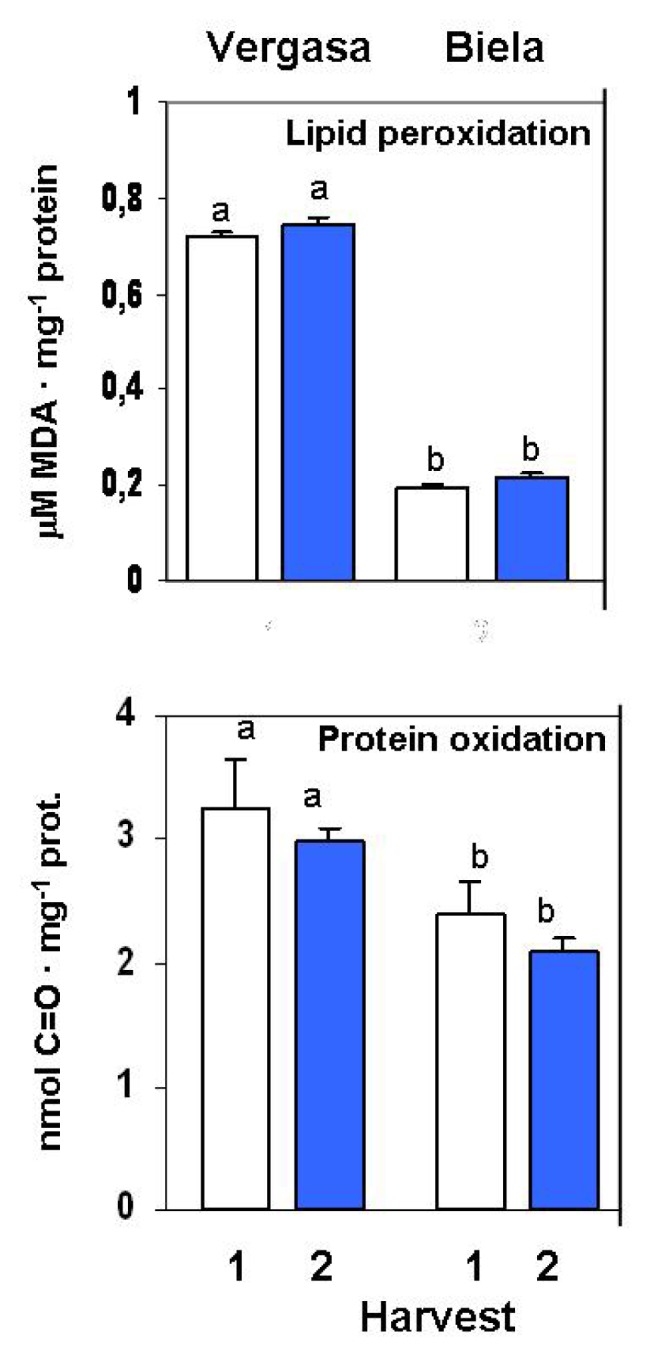
Lipid peroxidation and protein oxidation in cultivars Vergasa and Biela of pepper (*Capsicum annuum* L.) fruits harvested at two temperature conditions (Harvest 1 and 2). Lipid peroxidation was measured as μM MDA per mg protein, and protein oxidation as nmol carbonyl groups per mg protein. Column values with distinct letters are significantly different.

To investigate the possible imbalances in the oxidative metabolism of pepper fruits due to the temperature changes, the lipid peroxidation by the TBARS method and the protein oxidation as carbonyl groups content were determined. No differences among harvests were found in lipid peroxidation and protein oxidation in the two cultivars, with higher values of both parameters determined in Vergasa (Figure 8).

## 3. Discussion

Pepper fruit is a globally important vegetable crop for the fresh and processed food market. A huge diversity of varieties/cultivars is framed within the species *Capsicum annuum* with a number of phenotypic features (color, shape and flavor) and culinary properties, all of these sustained by distinct physiologies and metabolisms. During fruit setting, developing and maturation each cultivar may respond differently to adverse conditions generated by biotic and abiotic stressors, environmental factors or climate alterations, mainly temperature. Temperature is a key factor in the development and ripening of pepper fruits, with economical impact when crops undergo values below 15 °C [16–21]. The growing global demand of pepper fruits implies several strategies to increase crop production and fruit quality through specific agricultural fertilization practices [48] or promoting the investigation to improve the plant resistance to environmental stresses. In the absence of visual symptoms, the availability of internal parameters which may help breeders and companies to discriminate between the cultivars according to their response to environmental changes is one of the engines which drives the research of crop species due to its agronomic/economic impact. In this work a noteworthy increase of protein content was observed in fruits at Harvest 2 in both cultivars. This behavior could be of interest for crop purposes and deserves to be studied in a wider research.

The latest reports on the involvement of antioxidants in the pepper fruit physiology [5,21,49–56] prompted us to investigate the response of antioxidants from two sets of fruits which underwent different temperature profiles during development and ripening *in planta*, an approach which, to our knowledge, has never been boarded. Thus, a set of systems related to the ROS metabolism, including enzymatic and non-enzymatic antioxidants was analyzed in this work. The activity, protein content, and the gene expression through semiquantitative PCR of the enzymes CAT, SOD, APX, GR, GalLDH, G6PDH, 6PGDH, NADP-ICDH and EM were studied using actin as a constitutive gene which remains invariable during fruit ripening and development [52].

It is interesting to notice that the two California-type pepper fruits (Vergasa and Biela) showed similar profiles in most of the enzyme activities analysed, excepting catalase and glucose-6-phosphate dehydrogenase, throughout the experiment. This indicates that fruits from cultivars belonging to this pepper type seem to behave similarly under changing temperature conditions, independently on whether they differ in their ripening metabolism (*i.e.*, Vergasa fruits shift from green to red at ripening, whereas Biela does to yellow, what implies different carotenoid metabolism in their respective chromoplasts). The comparison of the results obtained suggested that the regulation of most enzymes investigated in pepper fruits, under the changing temperature conditions, might take place at a post-translational level. Whereas significant changes were detected in the activity of the enzymes from the cultivars investigated, little differences in the respective expression levels of the analyzed genes were detectable. In a recent work on the impact of two sanitized sewage sludges on the growth, yield, and fruit quality of pepper plants, the expression of the *AGC* and *GalLDH* genes was analyzed and, again, no changes were observed at the transcript level after those treatments [48]. Likewise, the expression of several leaf antioxidative genes (*Fe-SOD*, *Mn-SOD*, *CuZn-SOD*, *CAT*, *APX*, *MDAR*, and *GR*) was not modified by low temperature treatment of pepper plants [21]. Our results showed the great gene stability of the hybrid lines used in this work, proper of many other selected cultivars from diverse crop species which are usual in our gastronomy. However, since much of the enzyme systems investigated in this work are coded by several genes giving rise to distinct isozymes, much research is necessary to definitely assign the modulation of these genes to post-translational events.

In this work, we have observed that pepper fruits which developed at higher average temperature during pollination, fruit setting and maturation (October-November-December, Harvest 1) displayed greater reduced ASC content (and therefore, greater ASC/DHA ratio) than those developed in colder season (December-January-February, Harvest 2). The higher reduced ascorbate content in Harvest 1 observed in both cultivars could be due to an enhanced photosynthetic rate in fruits, since fruits from this set developed throughout longer days than those from Harvest 2. This hypothesis has been reported earlier in barley leaves, where the ascorbic acid content was dependent on the photosynthetic capacity [57]. The decrease in the reduced ascorbate in Harvest 2 correlates with an increased APX activity. On the contrary, the GR activity—which takes part in the ascorbate-glutathione cycle together with APX-decreased in Harvest 2 with respect to Harvest 1. This indicates that APX, apart from having a role in the ascorbate-glutathione cycle, might also act independently regulating the ascorbate redox state. The involvement of the AGC enzymes in other pathways and regulatory processes, besides their synergistic role in the H_2_O_2_ detoxification through the cycle, has been reported recently [41].

On the other hand, the levels of total ascorbate (reduced ascorbate + dehydroascorbate) were maintained basically constant in the two cultivars throughout the two harvests. Taking this into account and the very high ascorbate content of pepper fruits (about 0.1% FW), in the assayed conditions, a regulation role for ascorbate as a redox buffer which could contribute to the stability of the redox metabolism of pepper fruits is proposed, as it was previously postulated elsewhere [4,58]. Similarly, in a recent study, it has been reported that components of the ascorbate-glutathione cycle might play a role during pepper fruit ripening as modulators of plastid ASC and GSH redox states [55]. This stability of the total ascorbate content may be also responsible for the constant values observed in the TAA at the two harvests for the two cultivars, as it has been reported earlier where, besides ascorbate, some other compounds were assigned to contribute to the total antioxidant activity in pepper fruits, including flavonoids, capsaicinoids and polyphenols [5]. Due to the complexity of the metabolism of these last metabolites in pepper, a wider research is now been conducted in our laboratory in order to elucidate the specific role of each antioxidant in the whole picture of the fruit physiology.

In Harvest 2, a decline in the activity of GalLDH was detected with respect to Harvest 1, what indicated that, under those conditions, the regulation of the ascorbate levels might be altered. The GalLDH is sensitive to light, this enzyme being more active at higher sun radiation [59,60], as it actually takes place in Harvest 1. In studies performed earlier in isolated mitochondria from green and red pepper fruits from the cultivars Biela (California type) and Herminio (Lamuyo type), the GalLDH activity was unmodified what was postulated to be responsible for the maintenance of the ascorbate levels in both fruits [49]. In current analysis carried out in our laboratory on the expression level and enzyme activity of GalLDH and l-GalDH (l-galactose dehydrogenase), another enzyme of the ascorbate pathway synthesis, and the ascorbate content in different pepper organs (roots, shoots, leaves, flowers and fruits), results indicate that the modulation of such route depends on the cultivar and the organ (Mateos *et al.*, unpublished results). The discrepancy between the profiles of enzyme activity and gene expression suggests that both transcriptional and post-translational regulation may occur in our experimental conditions. However, more research is necessary to understand the molecular biology of the GalLDH in ripe pepper fruits.

NADPH is a key cofactor in cellular redox homeostasis, being an indispensable electron donor in numerous enzymatic reactions, biosynthetic pathways and detoxification processes. Besides being used in the photosynthetic Calvin-Benson cycle, NADPH is a necessary reducing equivalent for the regeneration of reduced glutathione by GR, and for the activity of the NADPH-dependent thioredoxin system, another important antioxidant [52,61]. Thus, the involvement of enzymatic components that regulate the production of essential antioxidant molecules such as GSH and NADPH indicates that the redox state of the cell is a cornerstone in the mechanism of regulation [62].

NADP-dehydrogenases mostly decreased at Harvest 2 and, consequently, the NADPH generation levels through these enzymatic systems. This suggests that the NADP-dehydrogenases studied in this work may be important components of pepper fruit physiology. Previous works have shown the involvement of different NADP-dehydrogenases in the mechanisms of response of pepper plants to high Cd concentrations [45], and in olive plants it was found that NADP-dehydrogenases are involved in the plant response to salinity [47]. Moreover, studies on some of these enzymes also showed the specific involvement of NADP-ICDH in leaf senescence [63] and this dehydrogenase also had a protective antioxidant role against certain environmental stresses [64]. More recently, in a research carried out in green and fully mature fruits from cultivars Vergasa and Biela, the involvement of the NADP-dehydrogenases in the pepper fruit ripening has been proposed [52].

Most of the ROS-related enzymes studied in this work as well as the ascorbate levels showed important differences by the effect of lowering temperature on developing and ripening fruits. Nevertheless, in spite of the changes observed in the ROS-related parameters, the analysis of both lipid peroxidation and protein oxidation, which are commonly used as indicators of oxidative stress [32,65–68] proves that fruits in the two cultivars do not undergo that syndrome. Thus, the parameters studied here could be used as tools for the investigation of pepper cultivars and fruit stability under the temperature changes, and this could be helpful for breeding and marketing purposes.

## 4. Experimental Section

### 4.1. Plant Material and Growth Conditions

**Figure 9:**
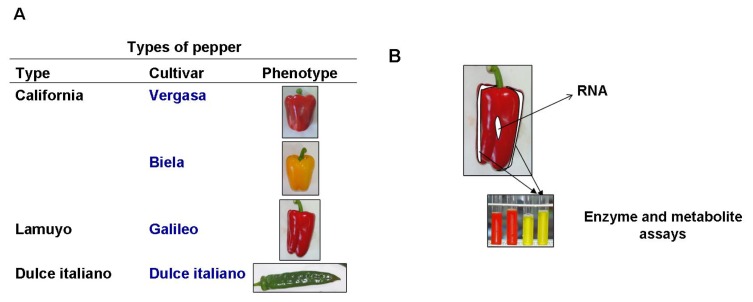
Experimental design for the analysis of ROS-related parameters in two cultivars of pepper (*Capsicum annuum* L.) fruits. (**A**) Types of pepper fruits used in this work belong to the cultivars Vergasa and Biela. Pictures of fruits correspond to the final stage in which fruits were analyzed: red for Vergasa and yellow for Biela; (**B**) Sampling of fruits for the analysis of enzymes and metabolites and gene expression. For enzymes and metabolite assays two strips per fruit were homogenized and analyzed. For RNA extraction the central part of the fruits was used.

Pepper (*Capsicum annuum* L.) fruits were obtained from Syngenta Seeds, S.A., El Ejido (Almería, Spain). Two cultivars Vergasa^©^ and Biela^©^ from type California were analyzed in this study. One noteworthy feature of these cultivars is their respective maturation colour. As seen in Figure 9, Vergasa becomes red at ripening, whereas Biela turns to yellow. Pepper plants from the two cultivars were cultivated in the same experimental greenhouse owned by Syngenta Seeds, S.A., according to the usual crop programme designed by the company: planting seeds and germination in July-August; flowering starts in late September and pollination and fruit setting takes places from middle October to late November. Meteorological data from these periods were collected from a station close to the experimental greenhouses and were recorded from October to February.

### 4.2. Experimental Design

**Figure 10:**
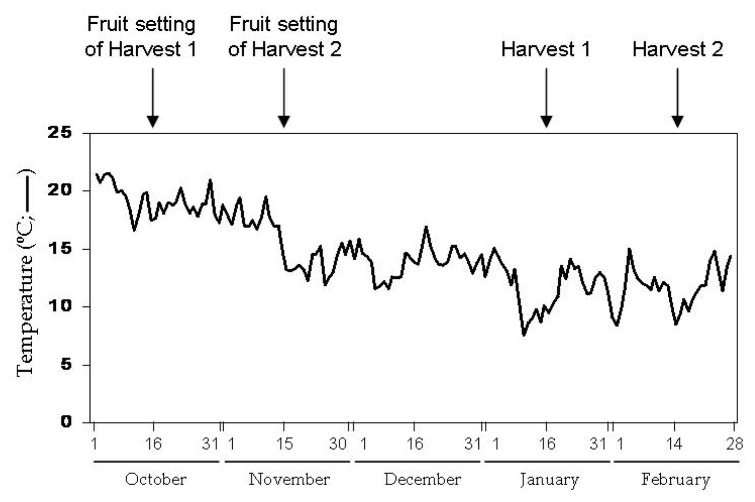
Temperature data corresponding to Harvests 1 and 2 of the present study. The data were provided by the Meteorological Station of La Mojonera (Almería, Spain) belonging to the *Junta de Andalucía*, Spain (UTM Coordinates *X*: 526472.0, *Y*: 4071536.0; latitude: 36°47′19″ N, longitude: 02°42′11″ W, altitude: 142.0 m).

In each cultivar, fruits were collected throughout the experiment from the same plants, randomly selected, in mid January (Harvest 1) and mid February (Harvest 2). These periods corresponded to fruit setting in mid October and mid November, respectively (aprox. 90 day intervals from setting to harvest in winter time; see Figure 10). Fruits from both harvests developed two different profiles of temperature with a continuous decline registered from the onset of the experiment (Figure 10). In fact, the average temperature for Harvest 1 including values from fruit setting (about October 15th) till fruit collection (January 15th) was 14.9 ± 0.3 °C. However, for Harvest 2 the average temperature (November 15th–Febraury 15th) was 12.4 ± 0.2 °C. External temperature influenced the conditions within the greenhouses as they do not contain either internal temperature control or sun screen except the glass cover of the facilities.

Ten different fruits were collected from ten plants (1 fruit/plant) of each cultivar Vergasa and Biela for each harvest, and all developed and ripened *in planta*. Two longitudinal strips were cut from each fruit (Figure 9): one of them was used for the analysis of ascorbate peroxidase, by further addition of ascorbate to the homogenization buffer (see below), and in the other strip the rest of enzymatic and non-enzymatic antioxidants, as well as other parameters such as lipid peroxidation, protein oxidation and total antioxidant activity were analyzed. Strips were frozen under liquid nitrogen and maintained at −80 °C until their analysis. In general, data corresponding to each parameter is the mean of, at least, five fruits.

For the RT-PCR analysis, one central piece from five fruits in each cultivar and each harvest was cut (Figure 9) and frozen at −80 °C until RNA was extracted.

### 4.3. Preparation of Crude Extracts

Fruit strips were weighed and homogenized at 4 °C in a mortar in the presence of 0.1 M Tris-HCl, pH 8.0, 0.1 mM EDTA, 2 mM DTT, 0.1% (*v*/*v*) Triton X-100, 10% (*v*/*v*) glycerol, in a ratio 1:2 (*w:v*). Homogenates were filtered through two layers of nyloncloth and centrifuged at 27,000× *g* for 15 min. Supernatants were used for the enzyme analyses. For the ascorbate peroxidase activity determination homogenates were prepared in the same buffer containing 2 mM ascorbate.

### 4.4. Enzyme Activities

Catalase (CAT; EC 1.11.1.6) was determined in 50 mM phosphate buffer, pH 7.0 by following the hydrogen peroxide breakdown at 240 nm as described by Aebi [69]. Superoxide dismutase (SOD; EC 1.15.1.1) activity was monitored by the method of McCord and Fridovich [70] based on the inhibition of the cytochrome c reduction by superoxide radicals (O_2_^·−^) generated by the system xanthine/xanthine oxidase. For the analysis of the isoenzymatic SOD pattern, sample proteins were separated by native PAGE on 10% acrylamide gels, and isoenzymes were visualized in gels by the NBT staining method [71]. SOD isozymes were detected in gels as achromatic bands over a blue background, consequence of the NBT reduction to formazan blue by the O_2_^· −^ radicals generated in the assay. The identification of the distinct isozyme nature, either CuZn-SOD, Mn-SOD or Fe-SOD was achieved by their specific sensitivity to the inhibitors KCN and H_2_O_2_: CuZn-SODs are inhibited by both, Fe-SODs only by H_2_O_2_, and Mn-SODs are resistant to both inhibitors.

Ascorbate peroxidase (APX; EC 1.11.1.11) was determined by monitoring the initial ascorbate oxidation by H_2_O_2_ at 290 nm [72]. To test the feasibility of the assay, 20 mM *p*-chloromercuriphenylsulphonic acid (*p*CMS), a specific inhibitor of APX, was used [73]. Glutathione reductase (GR; EC 1.6.4.2) was assayed by monitoring at 340 nm the NADPH oxidation coupled to the reduction of GSH [74]. The reaction rate was corrected for the small, nonenzymatic oxidation of NADPH by GSSG. The l-galactono-γ-lactone dehydrogenase (GalLDH; EC 1.3.2.3) activity was measured by the method reported by Ôba *et al.* [75] following at 550 nm the l-galactono-γ-lactone-dependent cytochrome c reduction.

For the analysis of the NADPH-generating dehydrogenases, glucose-6-phosphate dehydrogenase (G6PDH; EC 1.1.1.49), 6-phosphogluconate dehydrogenase (6PGDH; EC 1.1.1.44), isocitrate dehydrogenase (NADP-ICDH; EC 1.1.1.42), and malic enzyme (ME; EC 1.1.1.40), the procedures described earlier were performed [52]. These methods are based on the measurement at 340 nm of the NADPH production by the activity of the referred enzymes in the presence of glucose-6-phosphate, 6-phosphogluconate, isocitric acid and malic acid, respectively. Since G6PDH provides the substrate for the 6PGDH, in the assay of G6PDH, the enzyme activity of both G6PDH and 6PGDH were measured at once. The 6PGDH activity was determined using 6-phosphogluconate as substrate. Then, to calculate the G6PDH activity, the 6PGDH values were subtracted from the G6PDH assay.

Protein concentration in samples was determined by following the method of Bradford [76] with BSA as standard.

### 4.5. Western Blotting

SDS-PAGE was performed in 12% acrylamide slab gels (MiniProtean II, Bio-Rad Laboratories, Hercules, CA, USA), as described by Corpas *et al*. [44]. Prior to electrophoresis, samples were heated at 100 °C for 5 min in the presence of 0.1% (*w*/*v*) SDS and 5 mM DTT. Standards used were: phosphorylase b (M_r_ = 97,400), BSA (M_r_ = 66,000), ovoalbumin (M_r_ = 45,000), carbonic anhydrase (M_r_ = 31,000), soybean trypsin inhibitor (M_r_ = 21,500), and lysozyme (M_r_ = 14,400) (Bio-Rad Laboratories, Hercules, CA, USA). After SDS-PAGE, proteins were transferred to PVDF membranes (Immobilon P transfer membranes; Millipore Corporation, Bedford, MA, USA) in a Semi-Dry Transfer Cell (Bio-Rad Laboratories, Hercules, CA, USA). Membranes were processed for recognition using the immunoblot conditions described by Corpas *et al*. [44] with specific antibodies against Mn-SOD from pea leaves [77], Fe-SOD [78], CuZn-SOD from watermelon cotyledons [79], G6PDH from *Saccharomyces cerevisiae* [44], 6PGDH from spinach [80], and NADP-ICDH from pea [63,81].

### 4.6. RNA Isolation and Semiquantitative RT-PCR

Total RNA was extracted from the different pepper fruits using the Trizol method, and following the manufacturer’s instructions (GIBCOBRL, Life Technologies, Carlsbad, CA, USA). Samples (15 μg of total RNA) were subjected to electrophoresis on 1.2% (*w*/*v*) agarose-Mops gels under denaturing conditions [82]. Partial cDNAs for some of the analysed genes were obtained, confirmed by sequencing and deposited in the data bank (EMBL/GenBank) with accession numbers AY547351 for *GR*, AY547352 for *GalLDH* and AY173123 for *Fe-SOD*.

For the semiquantitative reverse transcriptase-polymerase chain reaction (RT-PCR) technique, the oligonucleotides included in Table 3 were used. Two μg of total RNA from leaves were used as a template for the reverse transcriptase reaction. It was added to a mixture containing 5 mM MgCl_2_, 1 mM dNTPs, 0.5 μg oligo (dT) primers, 1× RT-Buffer, 20 U Rnasin ribonuclease inhibitor, 15 U AMV reverse transcriptase (Promega, Madison, WI, USA). The reaction was carried out at 42 °C for 40 min, followed by a 5 min-step at 98 °C, and then by cooling at 4 °C.

Amplification of *actin* cDNA from pepper (EMBL/GenBank; AY572427) was chosen as a control. Each specific mRNA and *actin* cDNAs were amplified by polymerase chain reaction (PCR) as follows: 1 μL of the produced cDNA diluted 1/20 was added to 250 μM dNTPs; 1.5 mM MgCl_2_; 1× PCR buffer; 1 U of Ampli Taq Gold (PE Applied Biosystems, South San Francisco, CA, USA) and 0.5 μM of each specific oligonucleotide (see Table 2) in a final volume of 20 μL. Reactions were carried out in a Hybaid thermo-cycler. A first step of 10 min at 94 °C was followed by 28–33 cycles (depending on the enzyme) according to the following protocol: 30 s at 94 °C; 30 s at 60 °C; and 45 s at 72 °C. Amplified PCR products were detected by electrophoresis on 1% agarose gels and staining with ethidium bromide.

Quantification of the bands was performed with a Gel Doc System (Bio-Rad Laboratories, Hercules, CA, USA) coupled with a high sensitive CCD camera. Band intensities were expressed as relative absorbance units. The ratio between the specific enzyme and the *ACT* amplification was calculated to normalize the initial variations in sample concentrations [52,83]). Means and standard deviations (the latter not indicated in Figures) were calculated after normalization with *actin*. Thus, the numerical data reported in the panels of the expression analysis of the oxidative metabolism-related genes for each cultivar and at each harvest were calculated according to the expression displayed by the *actin*, which was used as the reference gene and was given value 1 in each condition, either cultivar or harvest.

### 4.7. Determination of Total Ascorbate Content and Total Antioxidant Activity

Basically, the method described by Jiménez and colleagues [84] was followed for ascorbate contents. Ascorbate was extracted from 0.5 g of pericarp fresh weight in 1 mL of 5% (*w*/*v*) meta-phosphoric acid with a mortar on ice. After incubation for 30 min at 4 °C, the mixture was diluted with distilled water to a final concentration of 2% (*w*/*v*) m-phosphoric acid, and was centrifuged at 12,000*g* for 10 min. ASC in the supernatants was determined immediately by HPLC following the absorbance at 254 nm, as described by Castillo and Greppin [85]. The total antioxidant activity was determined by the method of ABTS [ferrylmyoglobin/2,2′-azinobis-(3-ethylbenzthiazoline-6-sulphonic acid], by following either the absorbance decrease or the end-point products [86,87].

### 4.8. Lipid Peroxidation and Protein Oxidation

Lipid peroxidation was determined by measuring the concentration of thiobarbituric acid-reacting substances (TBARS) as described previously [88]. Assays were also performed in samples in the absence of thiobarbituric acid to avoid possible interferences by endogenous anthocyanins.

For the determination of carbonyl groups, the spectrophotometric dinitrophenyl hydrazine (DNPH) method of Levine *et al.* [65] was basically followed for each sample, using its respective blank. Samples containing at least 0.5 mg protein were incubated with 0.3% (*v*/*v*) Triton X-100 and 1% (*w*/*v*) streptomycin sulphate for 20 min to remove the nucleic acids, and were centrifuged at 2000*g*. Supernatants (200 μL) were mixed with 300 μL of 10 mM DNPH in 2 M HCl. The blank was incubated in 2 M HCl. After 1 h incubation at room temperature, proteins were precipitated with 10% (*w*/*v*) trichloroacetic acid and the pellets were washed three times with 500 μL of ethanol:ethylacetate (1:1). The pellets were finally dissolved in 6 M guanidine hydrochloride in 20 mM potassium phosphate at pH 2.3, and the absorption at 370 nm was measured. Protein recovery was estimated for each sample by measuring the *A*_280_. Carbonyl content was calculated using a molar absorption coefficient for aliphatic hydrazones of 22,000 M^−1^ cm^−1^ [89].

### 4.9. Statistical Analysis

The significance of differences between mean values obtained from the different independent experiments was determined by one-way analysis of variance. When the main effect was significant (*p* < 0.05) differences between means were evaluated for significance by the Duncan’s multiple range test.

## 5. Conclusions

Our results show that antioxidants from pepper fruits are involved in the response to temperature changes underwent due to local climatology. Pepper is a plant from tropical origin drastically affected by temperatures below 15 °C. However, in the conditions used in this work, with a set of fruits developing and ripening *in planta* at an average temperature of 12.4 °C, the antioxidative systems seem to compensate and buffer the imbalances generated under those unfavorable conditions. Thus, although great changes occur in the antioxidants from fruits subjected to temperature below 15 °C, no oxidative stress takes place as indicated by the stability of the lipid peroxidation and protein oxidation, two classic parameters associated to the oxidative stress syndrome. This pattern was observed in the two cultivars used in our experiments, Vergasa which ripens towards red fruits and Biela which does as yellow, but both framed within the same pepper type, California. The response observed in our work suggests that pepper fruits from California type respond to temperature changes issuing a canonical strategy which implies the involvement of cell antioxidants.

## Figures and Tables

**Table 1:** Total ascorbate (ASC + DHA), ASC/DHA ratio and total antioxidant activity (TAA) in cultivars Vergasa and Biela of pepper (*Capsicum annuum* L.) fruits harvested at two temperature conditions (Harvest 1 and 2). Values are means ± SEM.

	Vergasa		Biela	
		
Harvest	1	2	1	2
	
ASC + DHA (μg g^−1^ fruit)	1,118 ± 55	1,079 ± 160	1,267 ± 112	1,225 ± 223
ASC/DHA	1.41	0.61	1.36	0.59
TAA (μM)	3,656 ± 285	4,155 ± 125	4,410 ± 150	3,920 ± 127

**Table 2:** Protein concentration in cultivars Vergasa and Biela of pepper (*Capsicum annuum* L.) fruits harvested at two environmental conditions (Harvest 1 and 2). Values are means ± SEM.

	Protein concentration (μg/mL)	
	
Cultivar	Harvest 1	Harvest 2
Vergasa	340 ± 61	523 ± 76
Biela	144 ± 35	274 ± 55

**Table 3:** Oligonucleotides used for the cloning and semiquantitative reverse transcription-PCR analysis of the different antioxidative genes.

Enzyme	Oligonucleotide Sequences 5′ to 3′	Genebank Accession No
Catalase	F: GATTTCTTCTCTTTCCTCC R: CGATGTTCCTATTCAATACC	AF227952
Ascorbate peroxidase (cyt.)	F: TGTGCTCCTCTTATGCTCC R: CTCAAAACCAGAACGCTCC	X81376
Glutathione reductase	F: TTTGGTTTATGGAGCTGCC R: CAGTGGGAGTTGCTTTCTG	AY547351 *
Galactono-γ-lactone dehydrogenase	F: TTACTCTTCAGAACTTTGC R: GGATTGCATGTCACAACCAC	AY547352 *
Mn-superoxide dismutase	F: CATGCAGCTTCATCACCAGA R: ATAACAAGGCGCTTCAGCTC	AF036936
Fe-superoxide dismutase	F: CATCACAGGACCTATGTCG R: GGTGTTTTCACAACTACAAGC	AY173123 *
CuZn-superoxide dismutase	F: TGTTAGTGGCACCATCCTCT R: GGCCGATAATACCACAAGCA	AF009734
Actin	F: ACTCTTAATCAATCCCTCC R: GCACTGTATGACTGACACC	AY572427 *

F, forward; R, reverse;

* sequences obtained in this work; cyt, cytosolic.
